# Mechanisms of Pathology-Induced Neural Stem Cell Plasticity and Neural Regeneration in Adult Zebrafish Brain

**DOI:** 10.1007/s40139-018-0158-x

**Published:** 2018-01-16

**Authors:** Caghan Kizil

**Affiliations:** 1 German Centre for Neurodegenerative Diseases (DZNE) Dresden, Helmholtz Association, Arnoldstrasse 18, 01307 Dresden, Germany; 2 Center for Regenerative Therapies Dresden (CRTD), TU Dresden, Fetscherstrasse 105, 01307 Dresden, Germany

**Keywords:** Regeneration, Neurodegeneration, Neurogenesis, Adult zebrafish brain, Neural stem cell, Induced plasticity

## Abstract

**Purpose of the Review:**

The purpose of this study is to review the current knowledge on the damage-induced molecular programs that underlie the regenerative ability in zebrafish brain.

**Recent Findings:**

Neural stem cells are the reservoir for new neurons during development and regeneration of the vertebrate brains. Pathological conditions such as neurodegenerative diseases hamper neural stem cell plasticity and neurogenic outcome in humans, whereas adult zebrafish brain can enhance proliferation and neurogenic capacity of its neural stem cells despite the incipient pathology. Evidence suggests that zebrafish uses damage-induced molecular programs to enable neural stem cells to efficiently initiate regeneration. Since this aptitude may be harnessed for regenerative therapies in human brain, understanding the molecular programs regulating neural stem cell proliferation and quiescence in zebrafish is of utmost importance for clinical efforts.

**Summary:**

Specific molecular programs that are different than those in the homeostatic conditions regulate adult zebrafish neural stem cell plasticity and the regenerative capacity after injury and neurodegeneration. These programs can serve as candidates for stem cell-based regenerative therapies in humans.

## Introduction

Neural stem cells are the primary source for newborn neurons in vertebrates during development and regeneration [1–8], and their use in regenerative medicine is a motivation for designing novel therapeutic approaches. Therefore, understanding the molecular basis of neural stem cell plasticity and neurogenic ability in disease conditions is important for generating possible ways to harness such mechanisms for an efficient regenerative outcome in human brains.

During the development of the nervous system, radial glial cells that are derived from the neuroepithelium bear neurogenic ability [9]. These radial glial cells can give rise to all neuronal subtypes, and with subsequent maturation, patterning, and synaptic integration of the newborn neurons, the initial framework of the nervous system is established [10]. In mammals, during postnatal development, the majority of the radial glial cells transform into astrocytes, another type of glial cell, which assume considerably less neurogenic potential in vivo [10, 11]. A subset of astrocytes is set aside at least in two specialized stem cell zones, namely, the subventricular zone (SVZ) of the lateral ventricle and the dentate gyrus (DG) of the hippocampus, where constitutive adult neurogenesis endures [6, 12]. In some other vertebrates such as the teleost fish and urodele amphibians, the prevalence of neural stem cell distribution throughout the brain remains less restricted throughout their lives, and the neural stem cells retain their radial glial identity [2, 13–17]. For instance, in zebrafish, the neural stem cell proliferation zones are distributed along the whole rostro-caudal axis of the brain, and these regions are neurogenic throughout the life of the animal [2, 18–20]. Therefore, for questions addressing the neural stem cell plasticity and neurogenic capacity in relation to aging and pathology, adult zebrafish brain serves as an excellent vertebrate experimental system.

Mammalian and zebrafish neural stem cells respond differently to injuries and disease conditions. After traumatic injuries or stroke in mammalian brains, even though the neural stem cells increase their proliferation, the end readouts are inefficient neurogenesis and formation of a glial scar, which is a permanent structure of astrocytes that assemble around the lesion site and render the tissue restoration unfeasible [21–24]. On the contrary, traumatic injuries in adult zebrafish brain lead to increased proliferation of neural stem cells, do not induce glial scar, and therefore allow generation of new neurons that can integrate into the remaining circuitry [25–29]. A similar contrast between the regenerative capacity of zebrafish and mammalian brain can be observed after neurodegenerative conditions. In almost all neurodegenerative diseases in mammals, neural stem cell proliferation is reduced progressively, and when combined with the elevated levels of neuronal death, a substantial loss of brain mass takes place [30]. For instance, in several cerebral amyloidosis models that mimic Alzheimer’s disease in mice, neural stem cell proliferation severely decreases [31] in part due to the elevated levels of Amyloid-beta42—a small insoluble peptide cleaved from Amyloid precursor protein, and aggregates into toxic beta-sheet structures that constitute one of the major hallmarks of Alzheimer’s disease pathology [32, 33]. However, in zebrafish, although Amyloid toxicity causes symptoms of Alzheimer’s disease reminiscent of human brains (e.g., Amyloid-beta42 aggregation, cell death, inflammation, synaptic degeneration, and cognitive decline) [34, 35, 36•, 37••], neural stem cells in adult zebrafish brain can increase their proliferation and form new neurons that survive the toxicity and integrate into the circuitry [36•, 37••]. These findings indicate that the regenerative capacity of adult zebrafish brain is a result of induction of neural stem cell plasticity after neuronal damage, and understanding how zebrafish can elicit an efficient regeneration may help us to harness this “natural” knowledge to kick-start a similar neuro-regeneration response in human brains.

### Zebrafish Uses Damage-Induced Molecular Programs to Activate Neural Stem Cells

**Fig. 1:**
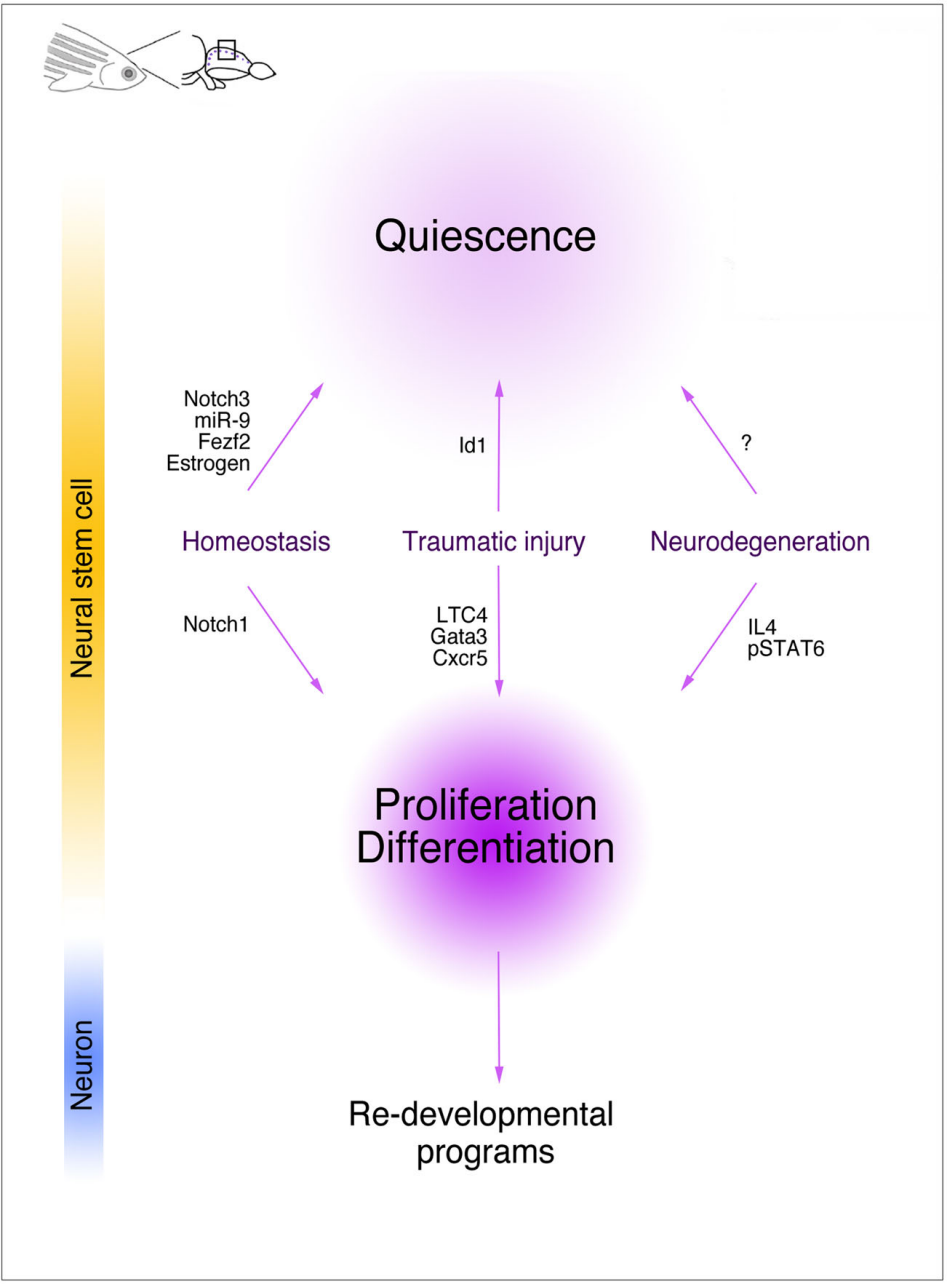
Schematic overview of distinct molecular programs regulating neural stem cell plasticity in adult zebrafish pallium during homeostasis, traumatic injury, and neurodegeneration. In homeostatic constitutive neurogenesis Fezf2 [50], Notch3 [49], miR-9 [53], and Estrogen [54] favor quiescence of neural stem cells where Notch1 [49] promotes proliferation Upon traumatic injuries, acute inflammation induces the plasticity of neural stem cells through LTC4 [42], Gata3 [41], and Cxcr5 [40] signaling, and Id1 [44] restores quiescence of neural stem cells. After Amyloid-mediated neurodegeneration, IL4 through phospho-STAT6 signaling is required for neural stem cell proliferation [37]. Please note that the programs required for neural stem cell plasticity in homeostatic conditions may also be employed after injuries or neurodegeneration; however, the programs in non-physiological conditions of injury and disease are specific to those damage paradigms and are not required for plasticity in homeostatic conditions. Redevelopmental programs are turned on for new neurons to differentiate and develop into respective cell types. For extensive reviews, please see [2, 13, 16, 19, 20, 29, 30, 39, 45, 46, 55]

Developmental and regenerative programs share common pathways and players [38]; however, the regenerative capacity seems to be dependent on a unique interpretation of the pathological environment in the brain and the type of neuronal loss by the neural stem cells [39] (Fig. 1). This hypothesis has supportive ground as different injury or disease models activate distinct molecular programs and crosstalk mechanisms between neural stem cells and various cell types including the microglia and neurons [36•, 37••, 39–43, 44••].

Traumatic injuries in adult zebrafish brain initiate an acute inflammation response involving pro-inflammatory cytokines and chemokines, which are in general detrimental for neural stem cell plasticity and regenerative ability in mammals [45, 46]. Interestingly, this innate immune response is positively regulating the neural stem cell proliferation in adult zebrafish brain at least through increasing the production of Leukotriene-C4 (LTC4) [42]. LTC4 is sufficient to activate the injury-dependent regeneration program by inducing the imminent expression of *gata3*, a transcription factor necessary for induced proliferation of neural stem cells after traumatic injury [41]. *cxcr5*, a chemokine receptor, also increases its expression after injury and is required for differentiation of proliferating neural stem cells to neurons [40].

Another study found that a Notch signaling-regulated basic helix-loop-helix protein *id1* is induced to its maximum expression levels at 5 days after traumatic lesion in adult zebrafish brain [44••]. *id1* is required for maintaining the quiescence of neural stem cells as morpholino-mediated knockdown increases; plasmid-based overexpression reduces the proliferation of neural stem cells. *id1* expression is not controlled by acute inflammation and is temporally anteceding *gata3* suggesting that *id1* is an injury-induced mechanism to balance the initial burst of neural stem cell proliferation by preventing the depletion of the neural stem cell pool [44••]. Other *id* genes in zebrafish might also be related to neural stem cell quiescence based on their expression patterns [43], while functional studies are needed to confirm these findings*.* Therefore, association of *id1* with quiescence during regeneration is a plausible hypothesis because by live imaging techniques developed for adult zebrafish brain [47••, 48••], injury was found to change the mode of neural stem cell division from asymmetric to symmetric [47••], which would in long term reduce the remaining stem cell population.

The synergistic effects of acute inflammation, *gata3*, and *id1* in initiating and balancing the injury-induced neural stem cell proliferation in adult zebrafish brain are independent of the homeostatic regulation of neural stem cell quiescence. This is based on the findings that the Notch signaling is a major determinant of stem cell quiescence [49], its gradient in the pallium is regulated by a transcription factor Fezf2 [50•], and microRNA miR-9 is a downstream effector of Notch signaling during development and homeostatic state of the adult fish brain [51, 52]. Furthermore, although the misexpression of Notch signaling components or miR-9 shifts the balance between proliferation and quiescence, these effects are independent of *gata3* expression [53••]. These findings suggest that adult zebrafish brain seems to have evolutionarily developed an intricate network of damage-induced molecular programs to supersede the developmental programs of neural stem cell plasticity and temporally adjust the proliferation-quiescence balance according to the high demand of neurogenesis after pathology.

The molecular basis of the regenerative neurogenesis response in neural stem cells after traumatic injury is also distinct from enhanced neurogenesis and rather relies on a complex set of cellular crosstalk to set the stage for the redevelopment of new neurons to take place [45]. A support for distinct regulatory mechanisms of neural stem cell plasticity during homeostasis and regeneration is the effects of estrogen, which reduces proliferation of ventricular progenitor cells only in a healthy brain while estrogen treatment does not affect neural stem cell proliferation after traumatic injury in adult zebrafish brain [54].

### Neurodegeneration Induces Interleukin-4-Mediated Neural Stem Cell Plasticity in Adult Zebrafish Brain

Several neurodegeneration models have been generated in zebrafish where expected pathological outcomes are observed. For instance, in larval zebrafish, various TAU models showed tangle formation and motor neuron degeneration in the spinal cord [34, 35, 56–61]. However, the effects of neurodegeneration on neural stem cells have not been addressed extensively. Recently, an Alzheimer-like model was generated in adult zebrafish brain by cerebroventricular injection [62–64] of monomeric human Amyloid-beta42, which caused toxic protein aggregation, neuronal death, synaptic degeneration, chronic microglial activation, inflammation, and impaired learning ability [36•, 37••]. Despite high Amyloid burden, adult zebrafish neural stem cells induce proliferation and subsequent neurogenesis in both young and old animals, suggesting a specific regenerative response. This response is mediated by the neurodegeneration-specific regulatory mechanism by cytokine interleukin-4 (IL4), which is both sufficient and necessary for induced neural stem cell plasticity [37••]. Production of IL4 is dependent on microglial cells but it is expressed mainly by the neurons containing Amyloid aggregations [37••]. IL4 initiates a signaling cascade in neural stem cells via its receptor *il4r* and induces the phosphorylation of intracellular effector STAT6; the transcriptional targets of which are currently unknown in adult zebrafish brain [37••]. Interestingly, although the neural stem cells in adult zebrafish brain induce their plasticity and neurogenic ability after traumatic injuries or neurodegeneration, they seem to do this by using divergent molecular programs. In the Alzheimer’s model of adult zebrafish brain, *gata3* expression is not induced, and similarly, traumatic lesions do not induce *il4* expression [37••]. These findings are interesting and suggest that neural stem cells of adult zebrafish brain respond by distinctive molecular programs to various pathologies; those programs are still different than the developmental programs, and understanding the molecular basis of such versatile regenerative responses may delineate particular therapeutic applications for human brains.

In a recent study, human TAU protein with a mutation (P301L) that generates one of the most aggressive forms of TAU aggregation and pathology in humans is chronically expressed using conditional cre/lox system in zebrafish brain [65••]. TAUP301L is known to follow the pathological route of Tauopathies by being hyperphosphorylated, forming oligomers, and in the end neurofibrillary tangles [60, 66]. Such a cascade has been shown in humans and also in zebrafish spinal cord in larval stages. Interestingly, although TAUP301L is hyperphosphorylated in adult zebrafish brain, it does not proceed in its pathological course to generate oligomers and neurofibrillary tangles [65••]. In this chronic expression model, expression of TAUP301L and its hyperphosphorylation does not lead to cell death, inflammation, increased proliferation of neural stem cells, and IL4 expression suggesting that zebrafish brain may have the potential to prevent Tauopathies. When the Amyloid-beta42 toxicity model was combined with chronic TAU expression in this model, neither the TAU expression exacerbated the Amyloid toxicity nor Aβ42 triggered neurofibrillary tangle formation [65••]. These results suggest that adult zebrafish brain engages discrete mechanisms to respond to neurodegenerative pathology. Additionally, neural stem cell plasticity and the molecular programs activated in neural stem cells can be used as prognostic markers of pathology and subsequent regenerative response in zebrafish, proposing an intriguing prospect of using zebrafish as a model to investigate how Tauopathies can be prevented in human brains.

### Conclusions and Open Questions

Neural stem cell plasticity is an important concept that underlies the ability of generating new neurons in response to damage. One of the major drawbacks of mammalian brains is the inability to induce neural stem cell proliferation and subsequent neurogenesis in an efficient way. Therefore, understanding the molecular basis of how zebrafish can perform well with its neural stem cells can open significant avenues for regenerative medicine.

Zebrafish taught us so far that even though the readout of different pathology models (e.g., traumatic injury, stroke, neurodegeneration) is increased proliferation of neural stem cells and elevated levels of neurogenesis, neural stem cells have their unique molecular signatures that lay the foundation for a particular type of regenerative response, which are different than the developmental and homeostatic programs of neural stem cell maintenance and plasticity [2, 37••, 39–42, 44••]. The importance of this finding is zebrafish brain can teach us which specific signaling cascade would be the most efficient way of inducing regeneration in diseased or compromised human brains. Valuably, these “damage-specific” molecular programs are likely to be the prime candidates for regenerative therapies.

Brain pathology has a complex course of development in humans. So far, no animal model could fully recapitulate the whole spectrum of the conditions in human brains, yet models provide reductionist approaches to particular aspects of diseases [31, 57, 67]. For this reason, the models generated in zebrafish should be validated for how well they resemble the human pathology by comparative histological and molecular analyses from patient materials. Furthermore, zebrafish’s promise could manifest when the molecular programs found in fish brain will be tested in mammalian systems. If those molecules that endow zebrafish neural stem cells the “regenerative ability” would prove useful to enhance the plasticity of neural stem cells in similar experimental models of mammalian brains, zebrafish will prove to be a useful model for serving as a shortcut for finding interesting candidate genes and proteins for therapeutic applications.

Neural stem cell proliferation and initial neurogenesis are important for providing the tissues a new reservoir of cells, but this is itself not enough for neuronal regeneration, which entails maturation and integration of differentiated neurons efficiently into the existing circuitry. Therefore, detailed studies on how newborn neurons survive, differentiate into certain subtypes of neurons, and integrate into the existing network should be performed in zebrafish brain. With existing drug-screening tools, imaging technologies, and genetic manipulation techniques [68–74, 75, 76•], zebrafish also offers a great promise for such investigations.
